# Supplementary material for: Complex‐centric proteome profiling by SEC‐SWATH‐MS
Source: Mol Syst Biol. 2019 Jan 14;15(1):e8438. doi: 10.15252/msb.20188438 (PMC6346213; doi:10.15252/msb.20188438)
Supplement: Supplementary file 8 — Dataset EV7 [file MSB-15-e8438-s008.zip › feature_plots_string/Q9H9B1.pdf]

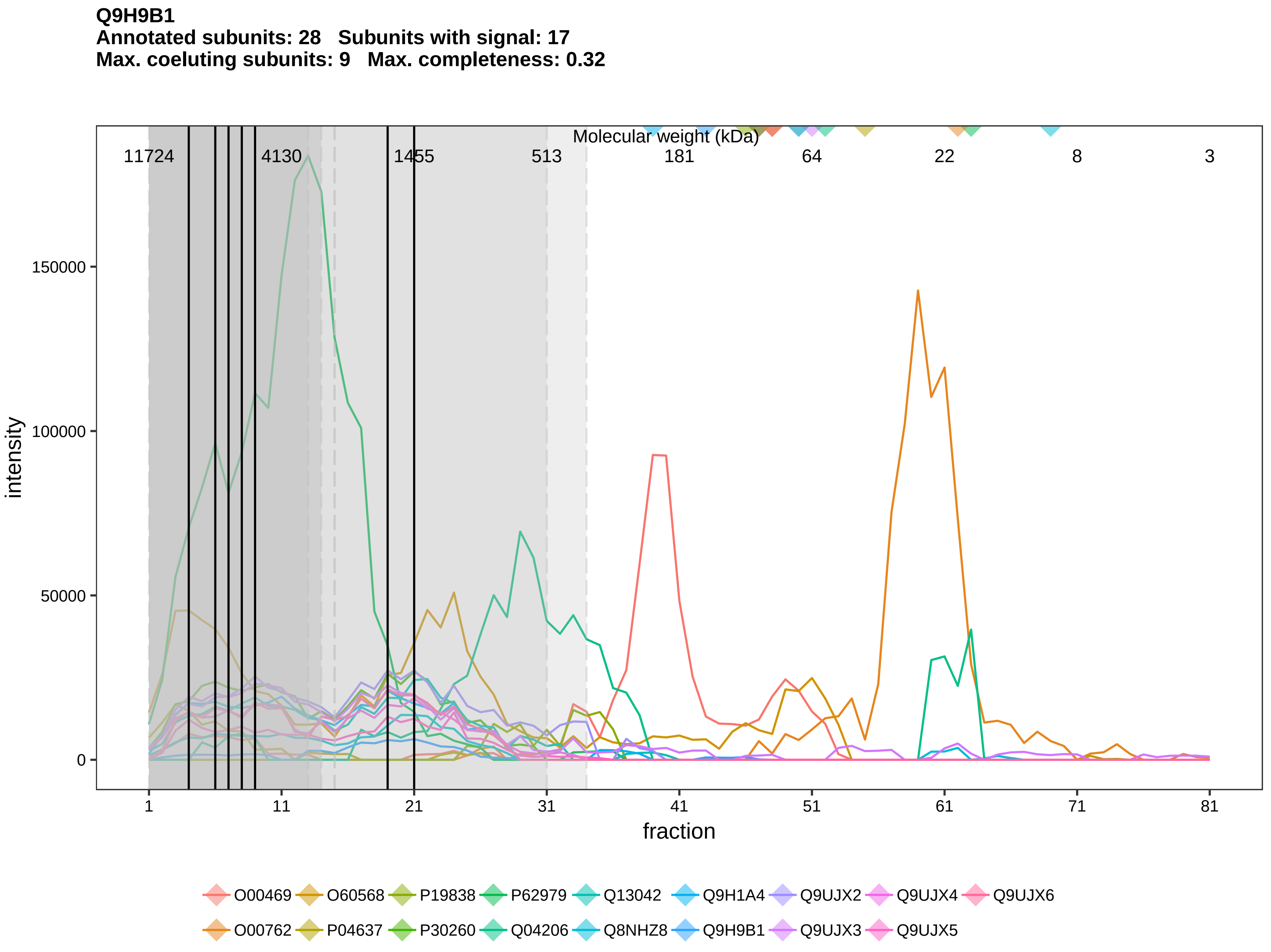

Q9H9B1
Annotated subunits: 28 Subunits with signal: 17
Max. coeluting subunits: 9 Max. completeness: 0.32
Molecular weight (kDa)
11724
4130
1455
513
181
64
22
8
3
intensity
150000
100000
50000
0
fraction
1
11
21
31
41
51
61
71
81
O00469
O60568
P19838
P62979
Q13042
Q9H1A4
Q9UJX2
Q9UJX4
Q9UJX6
O00762
P04637
P30260
Q04206
Q8NHZ8
Q9H9B1
Q9UJX3
Q9UJX5
